# Elevated gamma-glutamyl transferase is associated with subclinical inflammation independent of cardiometabolic risk factors in an asymptomatic population: a cross-sectional study

**DOI:** 10.1186/s12986-016-0097-7

**Published:** 2016-05-18

**Authors:** Shozab S. Ali, Ebenezer T. Oni, Michael J. Blaha, Emir Veledar, Hamid R. Feiz, Theodore Feldman, Arthur S. Agatston, Roger S. Blumenthal, Raquel D. Conceicao, Jose A. M. Carvalho, Raul D. Santos, Khurram Nasir

**Affiliations:** Center for Healthcare Advancement and Outcomes, Baptist Health Medical Group, 1691 Michigan Avenue Suite 500, Miami, FL 33139 USA; University of Manchester School of Medicine, Manchester, UK; Aventura Hospital and Medical Center, Aventura, FL USA; Department of Medicine, The Brooklyn Hospital Center, affiliate of Icahn School of Medicine at Mount Sinai, Brooklyn, NY USA; Johns Hopkins Ciccarone Center for the Prevention of Heart Disease, Johns Hopkins University, Baltimore, MD USA; Preventive Medicine Center, Hospital Israelita Albert Einstein, Sao Paulo, Brazil; Lipid Clinic-Heart Institute (InCor), University of Sao Paulo Medical School, Sao Paulo, Brazil; Robert Stempel College of Public Health, Florida International University, Miami, FL USA; Herbert Wertheim College of Medicine, Florida International University, Miami, FL USA

**Keywords:** Gamma-glutamyl transferase, C-reactive protein, Subclinical inflammation

## Abstract

**Background:**

Serum Gamma-Glutamyl Transferase (GGT), a marker of oxidative stress, has been suggested to be independently associated with cardiovascular disease (CVD) events. We examined the association of serum GGT levels with the burden of subclinical inflammation across a spectrum of metabolic conditions.

**Methods:**

We evaluated 5,446 asymptomatic subjects (43 ± 10 years, 78 % males) who had an employer-sponsored physical between 2008 and 2010. Highly sensitivity C-reactive protein (hsCRP) was measured as a marker of underlying systemic inflammation. A linear regression of GGT quartiles with log transformed hsCRP and a multivariate logistic regression of GGT quartiles with elevated hsCRP (≥3 mg/L) were performed.

**Results:**

Median GGT was 31 IU/l (IQR: 22–45 IU/l), 1025 (19 %) had hsCRP ≥ 3 mg/L. The median hsCRP increased with GGT quartiles (Q1: 0.9 mg/L, Q2: 1.1 mg/L, Q3: 1.4 mg/L, Q4: 1.6 mg/L, *p* < 0.001). Linear regression models showed GGT in the fourth quartile was associated with 0.45 mg/L (95 % CI 0.35, 0.54, *p* < 0.001) increase in log transformed hsCRP adjusting for risk factors. The Odds Ratio (OR) for an elevated hsCRP (≥3 mg/L) also increased with higher GGT quartiles; GGT Q2 1.44 (95 % CI 1.12, 1.85), GGT Q3 1.89 (95 % CI 1.45, 2.46), GGT Q4 2.22 (95 % CI 1.67, 2.95), compared to GGT Q1. The strength of association increased in the presence of and combination of metabolic conditions.

**Conclusion:**

In our cohort of asymptomatic individuals a higher serum GGT level was independently associated with increased burden of subclinical inflammation across metabolic states. These findings may explain GGT association with increased CVD risk.

## Background

Gamma-Glutamyl Transferase (GGT) is an enzyme present on the cell membranes of various tissues of the body; primarily the liver and also the kidneys, epididymis and lungs [1–3]. It is responsible for the extracellular catabolism of the antioxidant glutathione which leads to the production of reactive oxygen species (ROS). ROS play key roles in the inflammatory process and the progression of chronic diseases. Elevated GGT levels have been demonstrated to be associated with cardiovascular disease (CVD) risk factors including diabetes mellitus, hypertension, dyslipidemia and the metabolic syndrome [3–7]. It has also been linked with incident CVD [8, 9] and elevated levels have been found in atherosclerotic plaques [10]. Although some studies have demonstrated GGT as an independent marker of systemic inflammation and oxidative stress [11], its association with the CVD is still not clearly understood.

The highly sensitive C-reactive protein (hsCRP), an established marker of inflammation, has been demonstrated to be a predictor of myocardial infarction, stroke, peripheral arterial disease and sudden cardiac death [12–14]. Earlier studies have suggested that both GGT and hsCRP are associated with metabolic abnormalities [1], however the extent of the association of GGT as a marker for oxidative stress with hsCRP independent of cardiometabolic risks has not been clearly defined. This study examines the association of GGT distributed in quartiles with hsCRP as a measure of the burden of subclinical inflammation across a spectrum of the metabolic risk in a large asymptomatic cohort. We aim to assess the association of GGT with subclinical inflammation (hsCRP) and assess how this relationship is modified in varying states of metabolic health.

## Methods

### Subjects

We evaluated a group of 5,446 asymptomatic men and women, free of known cardiovascular disease, who submitted to routine clinical and laboratory health evaluation paid for by their employers from December 2008 to December 2010 at the Preventive Medicine Center of the Hospital Israelita Albert Einstein in São Paulo, Brazil. The examination protocol consisted of a clinical consultation, laboratory evaluation, and ultrasonographic abdominal scan. All individuals provided details of their demographics, medical history, quantitative alcohol consumption, smoking status, and medication usage at the time of their clinical consultation. Individuals with a known history of liver diseases were not included in the study.

### Measurements

Information regarding medical history was obtained via questionnaires. Physical activity level was assessed by a physical educator using the International Physical Activity Questionnaire: Short Form (IPAQ-SF), which has been previously validated in a similar patient population [15, 16]. Smoking status was defined as current smoker versus current non-smoker. Hypertension and dyslipidemia were ascertained by a previous history of these conditions or the use of blood pressure–lowering or lipid-lowering medications; those individuals with systolic blood pressure >140 mmHg or diastolic blood pressure >90 mmHg at the clinical evaluation were also labeled as having hypertension. Diabetes mellitus was identified by previous physician diagnosis or by the use of a glucose-lowering medication. Obesity was defined as a body mass index (BMI) >30 kg/m^2^ or >25 kg/m^2^ in individuals with a high waist circumference (WC). WC was defined as high if >94 cm in men or >80 cm in women. During physical examinations, blood pressure was measured with an aneroid sphygmomanometer using the American Heart Association recommended method [17]. WC was measured at the smallest diameter between the iliac crest and the costal margin using a plastic anthropometric tape held parallel to the floor. Alcohol consumption was quantified by the Alcohol Use Disorders Identification Test (AUDIT) score [18]. The AUDIT score was developed and validated by the World Health Organization among men and women in different countries. We categorized a total AUDIT score of ≥8 as high alcohol consumption for men and ≥ 4 for women [19]. Hepatic steatosis was diagnosed after at least a 6-h fast using an ACUSON XP-10 device (Mountain View, CA) and was identified by the presence of an ultrasonographic pattern of a bright liver, with evident contrast between hepatic and renal parenchyma, as previously described [20]. All hepatic ultrasounds were read by board-certified radiologists. The metabolic syndrome was defined using criteria from the American Heart Association/National Heart, Lung, and Blood Institute scientific statement on the metabolic syndrome [21]. Patients with ≥3 of the following metabolic risk factors were classified as having the metabolic syndrome: truncal obesity (≥102 cm [40 inches] for men and ≥88 cm [36 inches] for women), high blood pressure (blood pressure ≥130/85 mmHg or the use of antihypertensive medications), hyperglycemia (fasting blood glucose ≥100 mg/dL), low high-density lipoprotein cholesterol (HDL-C) (≤40 mg/dL for men and ≤50 mg/dL for women), and hypertriglyceridemia (≥150 mg/dL) [21].

Blood specimens were collected after an overnight fast. Plasma lipid, glucose, and liver transaminase levels (alanine aminotransferase [ALT] and aspartate aminotransferase [AST]) and Gamma-glutamyl transferase [GGT] levels were measured by standardized automated laboratory tests using a Vitros platform (Johnson & Johnson Clinical Diagnostics, New Brunswick, New Jersey). High-sensitivity CRP (hsCRP) levels were determined by immunonephelometry (Dade-Behring, GMbH, Mannhein, Germany). All tests were performed at the central laboratory of the Hospital Israelita Albert Einstein. This study was approved by the local institutional review board of Hospital Israelita Albert Einstein, and a waiver for informed consent was obtained [22]. All data was de-identified for statistical analysis.

### Statistical analysis

The baseline characteristics of individuals across the GGT quartiles were compared using the Pearson's χ^2^ test for categorical variables and ANOVA test for continuous variables. As a result of the skewed distribution of ALT, AST, and hsCRP, median values were compared using the non-parametric Kruskal-Wallis test. We log transformed hsCRP and conducted a multivariate linear regression analysis of lnhsCRP and GGT quartiles. We also evaluated the relationship of elevated hsCRP (≥3 mg/L) with GGT quartiles in a logistic regression model. An unadjusted analysis was done first followed by sequentially adjusting first for age and gender and then simultaneously adjusting for other confounding factors (waist circumference, triglyceride, HDL, SBP, fasting glucose, steatosis, LDL and smoking, alcohol consumption, ALT and AST levels). The association of GGT quartiles and hsCRP ≥3 mg/L in the presence of metabolic syndrome (MS), hepatic steatosis (HS) and across the combination of metabolic syndrome and hepatic steatosis was evaluated in a logistic regression analysis. All statistical analyses were performed using the STATA statistical software, release 12.

## Results

**Fig. 1:**
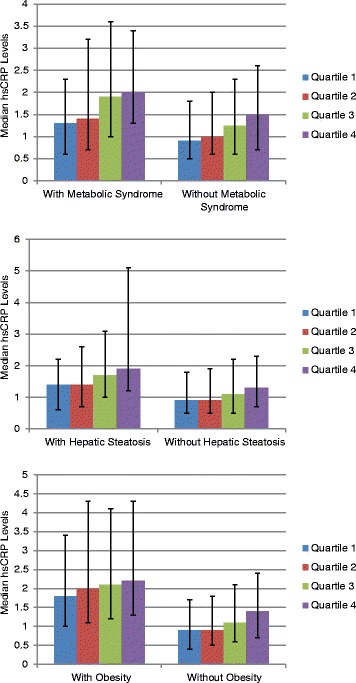
Relationship of median hsCRP levels with GGT quartiles across metabolic syndrome, hepatic steatosis and obesity. The median hsCRP levels increase across the GGT quartiles independent of the presence of metabolic syndrome, hepatic steatosis or obesity

**Fig. 2:**
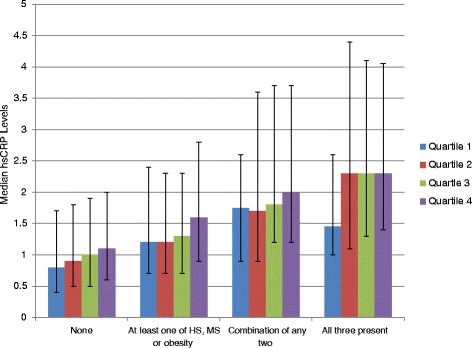
Median hsCRP levels in GGT quartiles across a combination of metabolic conditions; hepatic steatosis (HS), metabolic syndrome (MS) and obesity. The median hsCRP steadily increases with higher GGT quartiles as the number of metabolic conditions increases from none to three

The study population consisted of 5,446 asymptomatic, non-diabetic Brazilian subjects who were predominantly male (78 %) with an average age of 43 ± 10 years. The distribution of GGT was: overall median 31 IU/l (IQR: 22–45 IU/l) and cut off for the quartiles were; Q1: <22 IU/l, Q2: 22–31 IU/l; Q3: 31–45 IU/l; Q4: >45 IU/l. The clinical, anthropometric and biochemical characteristics of the study population across GGT quartiles are given in Table 1. Individuals with GGT in the fourth quartiles had the highest mean BMI, waist circumference, systolic and diastolic blood pressures (*p* < 0.001). They also were more likely to have higher levels of the other liver enzymes. While the median (IQR) hsCRP for the entire population was 1.2 (0.6, 2.4), it steadily increased with increasing GGT quartiles**.** Figure 1 demonstrates that this steady increase across the GGT quartiles is independent of the presence of MS, HS or obesity. Figure 2 shows the median hsCRP levels across GGT quartiles in the presence of a combination of hepatic steatosis, obesity and metabolic syndrome. The median hsCRP steadily increased with higher GGT quartiles as the number of metabolic conditions increased from none to three. A total of 1,008 (19 %) individuals had hsCRP > 3 mg/L, 1,979 (36 %) had hepatic steatosis, and 1,116 (21 %) had metabolic syndrome.

**Table 1:** Baseline characteristics of study participants across GGT quartiles

Characteristics	GGT-Q1 (0–22 IU/L)	GGT-Q2 (22–31 IU/L)	GGT-Q3 (31–45 IU/L)	GGT-Q4 (>45 IU/L)	*p*-value
	*N* = 1,465	*N* = 1,383	*N* = 1,254	*N* = 1,344	
Male (%)	46	80	89	95	<0.001
Mean age, y (±SD)	41(9)	43(10)	45(10)	45(9)	<0.001
Mean BMI, kg/m^2^ (±SD)	24(3)	26(4)	27(4)	28(4)	<0.001
Mean waist circumference, cm (±SD)	83(11)	90(11)	95(11)	98(10)	<0.001
Obesity, BMI ≥ 30 kg/m^2^ (%)	9	18	31	35	<0.001
Mean SBP, mmHg (±SD)	112(12)	118(12)	121(12)	124(13)	<0.001
Mean DBP, mmHg (±SD)	73(8)	76(7)	78(8)	80(8)	<0.001
HTN present(%)	6	11	15	19	<0.001
Antihypertensive (%)	6	10	14	19	<0.001
Median AST, IU/L (IQR)	25 (22,29)	28 (25,32)	30 (26,34)	34 (29,41)	<0.001
Median ALT, IU/L (IQR)	23 (19,29)	30 (25,38)	35 (29,45)	46 (35,61)	<0.001
Mean uric acid, mg/dl	4.8(1.3)	5.7(1.2)	6.2(1.2)	6.4(1.3)	<0.001
Median hsCRP, mg/L (IQR)	0.9 (0.5,1.8)	1.1 (0.6,2.1)	1.4 (0.7,2.7)	1.6 (0.9,2.9)	<0.001
Median triglycerides, mg/dl (IQR)	88 (67,119)	107 (79,149)	130 (95,180)	151 (108, 211)	<0.001
Mean HDL, mg/dl (±SD)	54(14)	49(13)	45(12)	45(12)	<0.001
Mean LDL, mg/dl (±SD)	120(30)	131(32)	134(34)	138(34)	<0.001
Mean FBG, mg/dl (±SD)	85(8)	88(9)	91(10)	93(13)	<0.001
NAFLD present(%)	9	27	49	59	<0.001
Statin (%)	4	7	9	12	<0.001
Current smoker (%)	7	8	10	11	<0.001
High alcohol consumption (%)	9	11	15	27	<0.001
Physically active (%)	80	79	73	70	<0.001

The adjusted linear regression analysis of the association between the GGT quartiles and log transformed hsCRP showed a steady increase in log transformed hsCRP moving from the second to fourth GGT quartiles, see Table 2. A logistics regression analysis, demonstrated that GGT levels in the higher quartiles was associated with higher odds of having elevated hsCRP (≥3 mg/L). The odds ratio (OR) of an elevated hsCRP increased with higher GGT quartiles from the second, third and fourth, OR 1.44 (95 % CI 1.2, 1.85), 1.89 (95 % CI 1.45, 2.46) and 2.22 (95 % CI 1.67, 2.95) respectively, compared to the first quartile, see Table 3. Table 4 shows the association between the higher GGT quartiles and elevated hsCRP across the spectrum of the metabolic states. The presence of these metabolic states is significantly associated with elevated hsCRP across higher GGT quartiles, the effect is attenuated with their absence.

**Table 2:** Linear regression analysis of GGT quartiles and lnhsCRP

	Model 1	Model 2	Model 3
Quartile 1 (ref)	1.0	1.0	1.0
Quartile 2	0.19 (0.11,0.26), *p* < 0.01	0.32 (0.24,0.39), *p* < 0.01	0.18 (0.10,0.27), *p* < 0.001
Quartile 3	0.45 (0.37,0.53), *p* < 0.001	0.61 (0.53,0.69), *p* < 0.001	0.32 (0.23,0.04), *p* < 0.001
Quartile 4	0.59 (0.52,0.67), *p* < 0.001	0.77 (0.69,0.85), *p* < 0.001	0.45 (0.35,0.54), *p* < 0.001

Model 1: unadjusted

Model 2: adjusted for age and gender

Model 3: model 2 + SBP, hdl-c, triglyceride-c, ldl-c, fasting glucose, waist circumference, physical activity, alcohol, AST, ALT, smoking, steatosis, statins

**Table 3:** Odds ratio of elevated hsCRP (≥3 mg/L) with GGT quartiles from a logistic regression analysis

	Model 1	Model 2	Model 3
Quartile 1 (ref)	1.0	1.0	1.0
Quartile 2	1.25 (1.02,1.54), *p* = 0.036	1.82 (1.45,2.27), *p* < 0.001	1.44 (1.12,1.85), *p* = 0.004
Quartile 3	1.74 (1.42,2.13), *p* < 0.001	2.86 (2.28,3.60), *p* < 0.001	1.89 (1.45,2.46), *p* < 0.001
Quartile 4	1.97 (1.62,2.40), *p* < 0.001	3.48 (2.76,4.39), *p* < 0.001	2.22 (1.67,2.95), *p* < 0.001

Model 1: unadjusted

Model 2: adjusted for age and gender

Model 3: model 2 + SBP, hdl-c, triglyceride-c, ldl-c, fasting glucose, waist circumference, physical activity, alcohol, AST, ALT, smoking, steatosis, statins

**Table 4:** The logistic regression of the presence of hsCRP >3 mg/L and GGT quartiles across a combination of metabolic syndrome and hepatic steatosis

	None *N* = 81	HS only *N* = 1,979	MS only *N* = 1,116	HS and MS *N* = 2,270
Quartile 1 (ref)	1.0	1.0	1.0	1.0
Quartile 2	1.64 (1.25, 2.15) *p* < 0.001	2.13 (1.07, 4.26) *p* = 0.03	1.19 (0.42, 3.38) *p* = 0.749	2.94 (1.05, 8.20) *p* = 0.04
Quartile 3	2.14 (1.56, 2.92) *p* < 0.001	3.34 (1.69, 6.62) *p* = 0.001	1.50 (0.54, 4.20) *p* = 0.440	3.88 (1.42, 10.60) *p* = 0.008
Quartile 4	2.43 (1.69, 3.50) *p* < 0.001	4.12 (2.07, 8.21) *p* < 0.001	1.72 (0.61, 4.86) *p* = 0.303	3.98 (1.45, 10.94) *p* = 0.007

Model was adjusted for age, gender, AST, ALT, statins, smoking

## Discussion

In this cross-sectional study of 5,446 healthy, non-diabetic Brazilian subjects, we found a significant relationship between GGT levels and hsCRP levels. Increasing GGT levels was associated with higher hsCRP levels across the entire spectrum of metabolic risk factors. After adjusting for possible confounding factors, the association persisted. Our findings suggest an association between GGT and subclinical systemic inflammation independent of cardiometabolic risk factors. The elevation in hsCRP with increasing quartiles of GGT may be a pointer to the role of higher GGT levels as a marker of cardiovascular risk [23]. This may help explain the association of increased oxidative stress measured by GGT and future cardiovascular events [24, 25].

GGT plays a crucial role in the extracellular catabolism of the antioxidant glutathione [24, 26], which facilitates the generation of ROS [12]. ROS have been implicated in early atherosclerosis. It is postulated that oxidative stress is the mediating factor of the association between GGT and cardiovascular disease [27]. However, it is unclear if GGT is induced as part of the antioxidant response to oxidative stress from inflammation associated with CVD or if GGT is a contributor to oxidant stress in atherosclerosis by generating ROS. Previous studies have demonstrated significant cross-sectional independent associations between serum GGT and CVD risk factors, like hypertension, stroke, and type 2 diabetes [3, 7, 23, 27, 28]. Liu et al. demonstrated the association of GGT and cardiometabolic risk factors was present both in a cross-sectional and in a prospective study of young healthy men [29].

Our findings indicate the association between GGT and hsCRP, an established marker for subclinical CVD, is independent of the metabolic conditions. This finding is supported by previous studies. Abdou et al. established an association between GGT and elevated levels of inflammatory biomarkers [15]. In another study, Bozbas et al., in a study of 232 patients (Mean age: 60 ± 10 years, 71 % females), demonstrated a significant correlation between GGT levels and CRP (*r* = 0.20, *p* = 0.003) mostly among individuals with metabolic syndrome [16]. In our study each of the metabolic conditions - metabolic syndrome, obesity or hepatic steatosis were associated with hsCRP; a combination of all these metabolic conditions was still associated with higher GGT quartiles.

This study demonstrated that the association between GGT and hsCRP presented a dose-response association. Lee et al., from the Coronary Artery Risk Development in Young Adults (CARDIA) study, showed a similar association. From their study, serum GGT predicted future concentrations of hsCRP in a dose–response manner [30]. In another large study from a representative sample of the US population using the third National Health and Nutrition Examination Survey (NHANES III), Lee et al. also demonstrated the association between serum GGT and CRP concentration across ethnic subgroups. They reported a strong association among all ethnic subgroups irrespective of the metabolic syndrome status [12], this further supports our findings. We have demonstrated for the first time that increasing GGT levels is associated with hsCRP independent of the metabolic states.

We must mention, that there have been some controversies regarding this association. In a recent study from the Prevention of Renal and Vascular End-stage Disease (PREVEND) study, the association of circulating GGT with CVD risk had comparable strength to risk of established cardiovascular risk factors [31]. While GGT showed a positive association with stroke risk in the model adjusted for traditional risk factors including CRP, the association with coronary heart disease (CHD) risk was abrogated when CRP was included in their model. The authors argued that elevated GGT levels could reflect chronic subclinical inflammation, often characterized by elevated CRP levels. As such CRP may be a confounder in the association between GGT and CVD [31]. Although the authors did not find the potential utility of GGT in CVD risk assessment, they support the argument that it plays a role in the etiology of CVD and potential causal relevance to CVD is important [31].

It is relevant to note that elevated levels of GGT in asymptomatic individuals can be due to many factors including but not limited to dyslipidemia, smoking, alcohol consumption, hypertension, hyperglycemia and, oral contraceptive use in women [32, 33]. Individuals with elevated GGT levels encountered in clinical practice with no liver dysfunction can be evaluated further for presence of subclinical inflammation. These individuals can be worked up for the presence of early atherosclerosis and may be considered candidates for more aggressive preventive strategies. This, however, needs to be studied further in future prospective cohort studies.

There are however some limitations to this study. Firstly, it is a cross-sectional study and as such causal relationships cannot be determined. Secondly, the study was performed in a cohort of Brazilian subjects which may limit the generalization. Also, the medical history was obtained from the subjects via questionnaire which is a potential source of recall bias. The use of ultrasound for diagnosing hepatic steatosis is limited by low sensitivity especially in obese individuals, and its inability to evaluate hepatic fibrosis. Another possible limitation is the “healthy worker effect” in the employee population.

The strength of this study lies in its large population size including participants of both genders, each undergoing extensive cardiovascular risk assessment. This allowed for adjustment for potentially confounding risk factors. Finally, our use of asymptomatic subjects may help make this data more relevant, as hsCRP measurement is performed commonly as part of a primary prevention strategy.

## Conclusion

In conclusion, this study demonstrates an association between GGT and elevated hsCRP levels among asymptomatic individuals, independent of obesity, metabolic syndrome and other cardiovascular risk factors. Additional research is however needed to elucidate the relationship between GGT and systemic inflammation as a measure for accurate prediction of future cardiovascular disease and the potential impact of risk reduction with therapies that lower GGT.

## Abbreviations

ALT: alanine aminotransferase
AST: aspartate aminotransferase
AUDIT: alcohol use disorders identification test score
CVD: cardiovascular disease
GGT: Gamma-Glutamyl transferase
HDL: high density lipoprotein cholesterol
HsCRP: highly sensitive C-reactive protein
HS: hepatic steatosis
IQR: inter-quartile range
LDL: low density lipoprotein cholesterol
MS: metabolic syndrome
ROS: reactive oxygen species
SBP: systolic blood pressure
